# Characteristics of Patients Reporting Presumed Problematic Drinking Behavior After Gastric Bypass: Exploring Long-Term Data From the BAROBS Study

**DOI:** 10.3389/fendo.2021.679006

**Published:** 2021-06-18

**Authors:** Magnus Strømmen, Christian Andreas Klöckner, Kirsti Kverndokk Bjerkan, Hallvard Græslie, Dag Arne Lihaug Hoff, Gjermund Johnsen, Bård Kulseng, Ronald Mårvik, Siren Nymo, Jorunn Sandvik

**Affiliations:** ^1^ Centre for Obesity Research, Clinic of Surgery, St. Olavs University Hospital, Trondheim, Norway; ^2^ Department of Clinical and Molecular Medicine, Norwegian University of Science and Technology, Trondheim, Norway; ^3^ Department of Psychology, Norwegian University of Science and Technology, Trondheim, Norway; ^4^ Faculty of Social Science and History, Volda University College, Volda, Norway; ^5^ Department of Surgery, Møre and Romsdal Hospital Trust, Ålesund, Norway; ^6^ Clinic of Surgery, Namsos Hospital, Nord-Trøndelag Hospital Trust, Namsos, Norway; ^7^ Department of Research and Innovation, Møre and Romsdal Hospital Trust, Ålesund, Norway; ^8^ Norwegian National Advisory Unit on Advanced Laparoscopic Surgery, Clinic of Surgery, St. Olavs University Hospital, Trondheim, Norway; ^9^ Obesity Research Group, Department of Clinical and Molecular Medicine, Norwegian University of Science and Technology, Trondheim, Norway

**Keywords:** gastric bypass, alcohol use and alcohol problems, RYGB, bariatric surgery, blackout

## Abstract

**Objective:**

To explore patients’ long-term experiences with drinking alcohol after Roux-n-Y gastric bypass (RYGB) for conceptualizing what may indicate problematic drinking behavior after bariatric surgery.

**Study Design:**

Three-center, observational study.

**Patients:**

546 adult patients undergoing RYGB in the period 2003-2009 in Norway.

**Main Outcome Measures:**

Self-reported data on drinking behavior and experiences related to alcohol collected 10-15 years after surgery.

**Results:**

Out of the 959 patients undergoing RYGB in the period, 29 were diseased and 546 participated in this follow-up study (58.7%). Focusing on suspicious changes in drinking behavior, 8.8% reported drinking more, 11.5% consumed alcohol at least twice a week, and 10.6% consumed at a minimum of 6 units of alcohol at a frequency of at least once monthly. The nature of hangovers had changed for about a third of the patients, with 21.6% reporting these to feel weaker or absent. Repeated alcoholic blackouts were reported by 11.9%. A subgroup of the patients were categorized as displaying presumed problematic drinking behavior(PPDB). Among the PPDB-men there was a significant association to having had a fall last year (6 (100.0%) PPDB-patients *vs.* 30 (29.7%) non-PPDB, p<.001). Among the PPDB-women, there was a significant association to having had alcohol problems prior to surgery (7 (70.0%) PPDB-patients *vs.* 67 (17.7%) non-PPDB, p<.001). Less significant associations to PPDB reported for explorative purposes were lack of patient education (men) (16 (26.2%) PPDB-patients *vs.* 8 (61.5%) non-PPDB, p=.014); more than 3 months persistent musculoskeletal pain (women) (45 (15.3%) PPDB-patients *vs.* 29 (24.6%) non-PPDB, p=.026); subjective problems with memory (women) (58 (20.7%) PPDB-patients *vs.* 10 (9.1%) non-PPDB, p=.006); and, receiving professional help for mental problems last 12 months (women) (29 (22.7%) PPDB-patients *vs.* 45 (14.7%) non-PPDB, p=.043).

**Conclusion:**

A subset of patients display drinking behaviors that may be consistent with postsurgical alcohol problems. Screening instruments like AUDIT may not be sufficiently specific to capture several risk behaviors occurring after bariatric surgery.

## Introduction

Several studies suggest that the Roux-n-Y gastric bypass (RYGB) is associated to increased risk of developing alcohol problems. Patients have reported that the feeling of intoxication appears earlier, even when consuming less alcohol, compared to before the operation (1). Seven years after RYGB, King et al. found a substantial increase in past year alcohol use disorder (AUD) symptoms when applying the Alcohol Use Disorders Identification Test (AUDIT). In addition, the number of patients reporting regular alcohol consumption (drinking ≥ 2 times/week) had doubled (2). Further, data from the Swedish Obese Subjects-study suggest that RYGB patients increased their daily alcohol intake after surgery above preoperative levels (3).

Increased consumption after RYGB is particularly concerning as ethanol bioavailability increases due to altered gastric first-pass metabolism and gastric transit time (4–6). Increased ethanol bioavailability means that even a continuation of the preoperative alcohol intake level could result in a considerably higher systemic ethanol exposure after the operation. This represents a pedagogical challenge when preparing patients for life after surgery. The effect RYGB has on alcohol absorption also complicates alcohol history taking and case detection for clinicians in the bariatric follow-up programs.

Another issue with increased ethanol bioavailability is that this may reduce the sensitivity of screening tools focusing on volume of alcohol intake. This applies to two of the questions in the AUDIT: “How many drinks containing alcohol do you have on a typical day when you are drinking?” and “How often do you have six or more drinks on one occasion?” Considering that one unit of alcohol will result in higher blood alcohol levels after RYGB, the relative weight on the response alternatives to these questions may be underestimated after surgery. Several studies have applied AUDIT on patients after bariatric surgery (2, 7, 8).

This study aims to provide a broad description of patients’ long-term experiences with drinking alcohol. We also try to conceptualize what we term *presumed problematic drinking behavior *(PPDB) after bariatric surgery. The last section is an explorative test for associations between a set of possible covariates and PPDB, which may offer an improved understanding of the validity of the PPDB-construct as basis for new hypotheses.

## Materials and Methods

All patients from three hospitals in Central Norway who underwent laparoscopic RYGB as treatment for severe obesity from 2003 to the end of 2009, were invited to a follow-up visit between August 2018 and June 2020. Out of the 959 patients in total, 29 patients were diseased and 546 patients chose to participate in a follow-up and thereby were included in the Bariatric Surgery Observation Study (BAROBS) (58.7%). The study was conducted according to the guidelines laid down in the Declaration of Helsinki. The study was approved by the Regional Committee for Research Ethics. All participants provided written consent.

The criteria for having RYGB were either BMI > 40 kg/m^2^ or BMI > 35 kg/m^2^ with obesity related comorbidities, as defined by national and international guidelines at the time.

The overall aim of the BAROBS study was to assess the general health status 10 to 15 years after RYGB as treatment for severe obesity. In addition, the study evaluated the long-term effect of RYGB on weight, resolution of comorbidities and quality of life, as well as drinking behavior. This paper addresses the latter.

### Statistical Analysis

The analysis strategy for this paper follows an exploratory path, which means that we compared the profiles of patients we categorized as potentially indicating problematic drinking behavior (hence showing at least 2 of our 6 indicators for problem drinking) on a high number of variables. In this analysis, we did not differentiate between predictors and outcomes of problematic drinking and we did not adjust for any confounders except for sex for which we ran the analyses separately. For the mapping of PPDB-patients against those without PPDB we used Chi^2^ tests for categorical variables and ANOVAs for continuous variables. Due to the high number of variables analysed and the corresponding alpha error inflation, we recommend the reader to be cautious when interpreting the significance levels indicated in **Tables 2** and **3** below. We have listed exact p-values as well as indicate p-values lower than.05 (conventional p-level) in addition to p-values adjusted for the number of tests.

### Operationalizing Problematic Drinking Behavior After Gastric Bypass

As we did not have a valid basis for categorizing the sample into patients with and without alcohol use disorder (AUD), we created a wider and more general category, aiming to identify patients that could be at risk of developing alcohol problems subsequent to RYGB. We applied the patients’ responses to a set of different questions that might indicate PPDB, seen in a post-surgical context. Six such indicator questions were identified in the data. These were patients reporting:

-consuming at least six alcoholic drinks on one occasion at least once monthly.
-ever been criticized by others because of their alcohol consumption.
-having had unpleasant experiences due to alcohol after the operation.
-reduced sensation of hangovers after the operation.
-consuming alcohol more frequently after the operation.
-having alcohol problems after the operation.

Patients showing two or more of these indicators were categorized as displaying PPDB.

## Results

Women accounted for 437 (80.0%) of the 546 participants. The mean (SD) age at the time of surgery was 40.4 (9.0) years. Mean observation time since surgery was 141 (19.0) months. BMI at referral for surgery was 46.5 (5.6) kg/m^2^.

### Self-Reported Drinking Behavior

With regard to alcohol problems, 41 patients (7.5%) reported to have experienced this after the operation. This compares to 14 patients (2.6%) reporting alcohol problems prior to the operation. A third of the patients reported their alcohol consumption had changed after the operation. While 142 patients (26.0%) said they either had stopped drinking alcohol, consumed less alcohol or drank less frequently, 48 patients (8.8%) reported drinking more often after the operation. About a third also reported that the nature of hangovers had changed. Hangovers were either weaker or completely absent after the operation for 21.6% of the sample, while 15.0% reported that the hangovers felt worse after the operation. See **Table 1** for data on alcohol consumption, data on whether patients received information about the possible effects on alcohol prior to surgery, as well as their experiences with blackouts and accidents.

**Table 1:** Self-reported data on drinking behaviour from 546 patients who had undergone Roux-n-Y gastric bypass.

ALCOHOL CONSUMPTION	Not at all the the last 12 months	Once a month or less frequently	2-4 times a month	2-3 times a week	4 times or more a week	Missing/Total
***How often have you consumed alcohol the last 12 months?***	99 (16.7%)	201 (36.8%)	165 (30.2%)	52 (9.5%)	11 (2.0%)	26/520
***How many alcoholic drinks do you normally consume during a two week period? (SD)***	4.0 (4.8) drinks					
	***Never***	***Less than once a month***		***Monthly***	***Weekly***	***Missing/Total***
***How often do you consume 6 or more alcoholic drinks on the same occasion?***	209 (38.3%)	162 (29.7%)		49 (9.0%)	9 (1.6%)	117/546
***REFLECTIONS ON CONSUMPTION***	***No***		***Yes***		***Missing/Total***	
***Have you ever felt that you should reduce your alcohol consumption?***	408 (74.7%)		93 (17.0%)		45/546	
***Have someone else ever criticized your alcohol consumption?***	446 (81.7%)		53 (9.7%)		47/546	
***Have you ever felt guilty because of your alcohol consumption?***	417 (76.4%)		82 (15.0%)		47/546	
***INFORMATION ABOUT EFFECTS OF SURGERY***	***No***		***Yes***		***Missing/Total***	
***Have you been informed that the effect of alcohol may change after gastric bypass?***	85 (15.6%)		425 (77.8%)		36/546	
***Were you informed prior to the operation?***	75 (13.7%)		321 (58.8%)		150/471	
***Were you informed after the operation?***	61 (11.2%)		310 (56.8%)		175/546	
***Were you informed by health personnel?***	39 (7.1%)		339 (62.1%)		168/546	
***Were you informed by other patients or others?***	196 (35.9%)		168 (30.8%)		182/546	
***Have you had unpleasant experiences related to alcohol after the operation?***	345 (63.2%)		151 (27.7%)		50/546	
***BLACKOUTS AND ACCIDENTS***	***No***		***Only once***		***Repeatedly***	***Missing/Total***
***Have you experienced alcohol blackout after the operation?***	26 (4.8%)		57 (10.4%)		65 (11.9%)	398/546
	***During the last 4 weeks***		***1-12 months ago***		***More than a year ago***	***Missing/Total***
***When was the last time you had a blackout?***	13 (2.4%)		41 (7.5%)		73 (13.4%)	419/546
***How many alcoholic drinks had you consumed when the blackout set in?***	6.3 (4.6) drinks					
	***No***		***Yes, but suffered no injuries***		***Yes, and had to see a doctor***	***Missing/Total***
***Have you after the operation fallen while being under the influence of alcohol?***	80 (14.7%)		59 (10.8%)		14 (2.6%)	393/546

Data collected 10-15 years after the operation.

### Differences Between Men and Women

Men and women did not differ in terms of age at the time of surgery or highest BMI as measured at the hospital prior to the operation. However, with regard to alcohol, men reported significantly higher alcohol consumption than women: Men drank nearly twice as much as women over a two-week period, and more men than women reported drinking six or more alcoholic drinks at the same occasion at least monthly. Men also reported having consumed a considerably higher number of drinks when experiencing alcoholic blackouts. See **Table 2** for details.

**Table 2:** Age at time of surgery, maximum BMI before surgery, and self-reported data on drinking behaviour 10-15 years after surgery: Comparison of data from men and women.

	Men	Women	Test-statistic	*p*
***Age at surgery (years (SD))***	41.3 (9.4)	40.2 (8.9)	*F*_1,546_=1.338^a^	.248
***Highest BMI (kg/m^2^ (SD))***	47.4 (6.8)	46.3 (5.2)	*F*_1,546_=3.448^a^	.064
***DRINKING BEHAVIOUR***				
***Number of alcoholic drinks normally consumed in 2 weeks (SD)***	6.2 (6.9)	3.4 (3.9)	*F*_1,408_=24.670^a^	<.001*
***Number of alcoholic drinks consumed when experiencing blackout (SD)*** *^†^*	9.5 (6.1)	5.5 (3.8)	*F*_1,107_=14.467^a^	<.001*
***Consume 6 or more alcoholic drinks on the same occasion at least once monthly***	24 (26.4%)	34 (10.1%)	χ^2^(1,N=429)=16.321^b^	<.001*
***Consumed alcohol at least 2 times weekly last 12 months***	23 (21.5%)	40 (9.7%)	χ^2^(1,N=520)=11.132^b^	<.001*
***Felt guilty because of your alcohol consumption***	23 (21.7%)	59 (15.0%)	χ^2^(1,N=499)=2.717^b^	.099
***Have experienced repeated blackouts due to alcohol after the operation*** *^†^*	13 (44.8%)	52 (43.7%)	χ^2^(1,N=148)=0.12^b^	.912
***Have fallen when being influenced by alcohol*** *^†^*	19 (61.3%)	54 (44.3%)	χ^2^(1,N=153)=2.873^b^	.090
***Have at least 2 indicators of problematic drinking behavior***	36 (33.0%)	74 (16.9%)	χ^2^(1,N=546)=14.046^b^	<.001*

SD, standard deviation; ^a^F-test (ANOVA); ^b^χ^2^=Pearsons Chi-square; *Significant at the .005-level (adjusted for total number of tests); ^†^ Note small N due to requiring positive answer to other question.

### Characteristics of Patients Reporting Presumed Problematic Drinking Behavior After Gastric Bypass

Patients were categorized either as displaying PPDB after RYGB or not displaying such behavior. See the Method section for the list of indicator variables of which two or more had to be present in order to fall in the PPDB category.

Among men with PPDB there was a significant association to having had a fall the last year. There were also indicative data suggesting that patient education about the possible effects of RYGB on alcohol could be protective for developing PPDB.

None of these associations were observed among women. In contrast to men there was a significant association between having had alcohol problems prior to the RYGB among women with PPDB. Other associations to PPDB in women were having at least three months of persistent musculoskeletal pain; reporting problems with memory; and, receiving professional help for mental problems at some point during the last 12 months. However, these associations were less significant and primarily indicative.

Other variables that were tested for associations to PPDB and found to be non-significant were having experienced physical, psychological or sexual abuse; cohabitation; having broken out from a relationship after the operation; smoking tobacco; had a bone fracture after the operation; and, reporting working out regularly. Details are listed in **Table 3**.

**Table 3:** Testing different variables for possible associations to presumed problematic drinking behaviour (PPDB) reported after Roux-n-Y gastric bypass.

	Men				Women			
Variable	Yes (PPDB)	No (no PPDB)	Test^a^	*p*	Yes (PPDB)	No (no PPDB)	Test^a^	*p*
***Persistent musculoskeletal pain the last three months***	16 (31.4%)	19 (35.8%)	χ^2^(1,N=104)=.233	.629	45 (15.3%)	29 (24.6%)	χ^2^(1,N=413)=4.980	**.026^#^**
***Received information prior to surgery about its effect on alcohol***	16 (26.2%)	8 (61.5%)	χ^2^(1,N=74)=6.097	**.014^#^**	47 (18.1%)	17 (27.4%)	χ^2^(1,N=322)=2.744	.098
***Experienced trauma (physical, psychological or sexual abuse)***	8 (40.0%)	28 (31.5%)	χ^2^(1,N=109)=.538	.463	34 (18.5%)	40 (15.8%)	χ^2^(1,N=439)=.539	.463
***Cohabitant or married***	22 (30.1%)	14 (38.9%)	χ^2^(1,N=109)=.835	.361	49 (17.0%)	25 (16.8%)	χ^2^(1,N=437)=.004	.950
***Had alcohol problems before the surgery took place***	2 (50.0%)	34 (33.7%)	χ^2^(1,N=105)=.456	.500	7 (70.0%)	67 (17.7%)	χ^2^(1,N=388)=17.249	<.001*
***Smoking tobacco***	8 (38.1%)	28 (32.6%)	χ^2^(1,N=107)=.232	.630	26 (23.4%)	48 (15.9%)	χ^2^(1,N=413)=3.129	.077
***Reporting problems with memory***	24 (39.3%)	9 (25.7%)	χ^2^(1,N=96)=1.831	.176	58 (20.7%)	10 (9.1%)	χ^2^(1,N=390)=7.412	**.006^#^**
***Had a bone fracture after the operation***	6 (35.3%)	12 (35.3%)	χ^2^(1,N=51)=.000	1.000	19 (22.1%)	8 (10.7%)	χ^2^(1,N=161)=3.747	.053
***Had a fall the last year***	6 (100.0%)	30 (29.7%)	χ^2^(1,N=107)=12.536	<.001*	13 (18.3%)	60 (17.6%)	χ^2^(1,N=411)=.018	.894
***Working out weekly***	27 (35.5%)	9 (29.0%)	χ^2^(1,N=107)=.416	.519	60 (18.8%)	14 (15.1%)	χ^2^(1,N=413)=.669	.413
***Have had professional help for mental problems the last year***	6 (35.3%)	30 (32.6%)	χ^2^(1,N=109)=.047	.829	29 (22.7%)	45 (14.7%)	χ^2^(1,N=435)=4.093	**.043^#^**
***Broken out of relationship after the operation***	8 (26.7%)	28 (35.4%)	χ^2^(1,N=109)=.757	.384	17 (18.9%)	57 (16.4%)	χ^2^(1,N=437)=.308	.579

a Pearsons chi square. *Significant at the .004-level (adjusted for number of analyses). ^#^Significant at the .05-level (not adjusted, explorative purpose).

The patients underwent gastric bypass 10-15 years earlier. Separate analyses for men and women.

## Discussion

This study investigates drinking behavior among RYGB patients 10 to 15 years after their operation. In terms of presurgical global demographics provided by the International Federation for the Surgery of Obesity (IFSO) (9), the BAROBS-sample patients were slightly younger (40 *vs.* 42 years) and had a higher median BMI (46.0 *vs.* 41.7 kg/m^2^). There were also a higher proportion of women (80.4 *vs* 73.7%) in the Norwegian sample compared to the numbers reported by IFSO.

### Patients’ Experiences With Blackouts

The paper attempts to give a broad description of patients’ drinking behavior after RYGB. While some of the questions reflect concepts from the AUDIT used in the screening for alcohol use disorders, other questions mirror our own experiences with the patients after RYGB. An example of the latter are the questions addressing the nature of early alcoholic blackouts, a phenomenon that reflects the increased bioavailability of ethanol. Early blackouts became a qualitatively new experience for a subset of the patients and may be essential for understanding the risks these patients face after surgery, both linked to injuries and the development of an addiction. In particular, patients reporting repeated experiences of blackouts, may be at increased risk of developing an alcohol problem subsequent to surgery.

Applying solely AUDIT with the standardized scoring algorithm in this population does not account for the risks related to faster and increased alcohol absorption. This suggests a need for adjusting the weighting of the AUDIT-items pertaining to alcohol consumption after RYGB and, perhaps also, including a subset of questions more directed towards the altered pharmacokinetics of ethanol following RYGB.

### Defining Problematic Drinking Behavior in a Postsurgical Context

When aiming to describe what characterizes patients with PPDB it is compelling to ask whether we have identified the patients of interest. First of all, the BAROBS study aimed at describing health outcomes broadly after RYGB. Consequently, the questions on alcohol were just a small part of a comprehensive battery of questions. In addition, the size of the BAROBS sample ruled out the option of diagnostic interviews for AUD. Furthermore, as screening instruments such as the AUDIT are not adapted to the altered pharmacokinetics of ethanol after RYGB, its recommended threshold for problematic alcohol use may be misleading for this population. However, the BAROBS questions on alcohol may serve explorative purposes, and we focused on six different questions on drinking behavior that potentially could be indicators of PPDB for the bariatric surgery patient.

The first of the six indicator questions refers to a consumption of at least six alcoholic drinks on a single occasion at least once monthly (n=24), and mirrors the third question in the AUDIT. Deeming six drinks to represent a significant consumption for people in general, we also need to take into account the heightened systemic alcohol exposure patients may experience after RYGB. Thus, linking such volumes of consumption to frequency, ‘at least once monthly’, justified this as a potential indicator of PPDB. An apparent limitation to using a certain volume to define PPDB may also be sex differences in the ability to metabolize alcohol.

The second question, ‘ever been criticized by others because of your alcohol consumption’ (n=19), is similar to the last question of the AUDIT. It seems reasonable that one may reach a significant level of consumption before others take the burden of confronting the patient. Thus, this may be an indicator of PPDB.

The third operationalization of PPDB was linked to those reporting consuming alcohol more frequently after the operation (n=15). Although the lack of a reference value, this question captures patients with a relative increase in alcohol consumption. One may assume that among adults with no alcohol problems, the frequency of alcohol consumption is somewhat stabile over time.

The fourth question was ‘having had unpleasant experiences due to alcohol after the operation’ (n=31). Unpleasant experiences may be considered either too vague or too general, and this question would not be useful if patients understood it to refer to a common hangover. However, if the question reflects more serious experiences the patient regrets it may signal a loss of control when drinking.

The fifth question aimed at identifying those who experience their hangovers less burdensome after the operation (n=26). This question relates to our clinical experience with patients with alcohol problems after bariatric surgery. Many have reported hangovers either to be less burdensome or become absent after the operation. As the hangover represents a negative physiological feedback on excessive drinking, we expect those who lose this mechanism to become more prone to alcohol problems after surgery.

The final indicator question was whether they had experienced alcohol problems after the operation (n=13). Although patients may consider the criteria for alcohol problems differently, this very direct question can identify patients who have come to realize that their drinking pattern is hazardous. While the other PPDB-questions focus solely on a particular behavior, admitting to having an alcohol problem is a direct question that involves the cognitive processing of one’s own behavior and experiences. Hence, the lower number of patients responding positively to this question compared to the other PPDB-questions may be expected. Also worth commenting is that the proportion of patients reporting alcohol problems after the operation should not be compared to the group reporting this prior to the surgery. Besides problems relating to recall bias, patients admitting to have alcohol problems in the referral process for RYGB may have been rejected surgery.

The number of patients who responded positively to the PPDB-questions varied from 13 to 31. There was low consistency in how patients responded to the six PPDB-questions, i.e. while 138 patients responded positively to one of the six questions, all six indicators were present only in 5 patients. This suggests that the concept of PPDB after bariatric surgery may be multifaceted and our questions tap into qualitatively different aspects of PPDB. However, the few patients confirming several PPDB-questions also raises doubt whether responding positively to only one of the six questions really mirrors the phenomenon of interest. Consequently, to be categorized in the PPDB-group we required a minimum of two positive responses on the PPDB-questions. This produced a PPDB-group of 59 patients.

### Differences Between Men and Women

Men and women differed significantly in terms of self-reported alcohol consumption with men consistently reporting higher and more frequent alcohol intake compared to women. Such a pattern may be expected as men on average drink more than women (10). Men and women also differ in terms of alcohol pharmacokinetics (11).

The magnitude of the observed differences between men and women suggests that sex may be a confounder in the explorative analyses for associations to PPDB. Consequently, we chose to run the analyses for associations separately for men and women.

### Characteristics of Patients Reporting Problematic Drinking Behavior

The BAROBS study consists of a substantial collection of data on Norwegian patients that underwent RYGB 10 to 15 years earlier (2003-2009). This paper explores eventual associations between PPDB and a selection of these data. The variables we chose to test for associations to PPDB were information that theoretically either could contribute to vulnerability for developing alcohol problems (e.g. patients having experienced trauma) or could represent negative health outcomes after surgery for which alcohol may have played a contributing role (e.g. patients reporting falling). This study tests altogether 12 variables for eventual associations. See **Table 3** for details.

#### Smoking

There is a known and strong positive association between smoking and drinking alcohol (10, 12). Although there were higher proportions of both male and female smokers among the patients with PPDB, our data does not confirm any association between PPDB and smoking. Factors potentially influencing the prevalence of smoking in our sample are that they are recommended to cease smoking before surgery, smoking cessation is a condition for plastic surgery after weight loss, and the follow-up after bariatric surgery involves a focus on healthy lifestyle. Furthermore, Norway has imposed strong public health measures to reduce smoking, with the proportion of Norwegians smoking reduced by more than 50% over the last ten years (13). Altogether, this may weaken any association between PPDB and smoking.

#### Musculoskeletal Pain

Patients reported whether they had had persistent musculoskeletal pain the last three months. Such pain is common and may be of intermittent or chronic nature. As alcohol may be a coping mechanism for patients particularly with chronic pain, we expected to find an association between PPDB and musculoskeletal pain. For men there was no such association, while there was an indication that women with musculoskeletal pain show less PPDB. Although this finding was contrary to our expectations, it is actually in line with data both from a Danish epidemiological study (14) and a Swedish matched control study (15) which found alcohol use to be less common among chronic pain patients.

#### Traumatic Experiences

There is a strong relationship between adverse childhood experiences and adult alcohol misuse (16). The variable we tested was not limited to experiences in childhood, it encompasses abuse of both physical, psychological and sexual character at any time in life. Despite a number of patients reported having traumatic life events, we did not find any statistical association between PPDB and such experiences.

#### Living Together – And Breaking Up

Whereas the home may be an arena for abuse, we assumed that patients living together with someone could be a protective factor against PPDB. When identifying patients living together with someone as opposed to living alone, we did not differentiate between cohabitation and married patients. There was, however, no association between living together with someone and PPDB for men as well as for women. It could be that as we did not differentiate between married and cohabitating patients we concealed that these two forms for relationships may differ in terms of stability and support. It has been found that cohabitation is associated to higher prevalence of heavy episodic drinking compared to marriage (17).

We also tested associations between PPDB and breaking up from a relationship. Since divorce statistics have been found to increase after bariatric surgery (18) and divorce and widowhood is associated with risk for alcohol use disorder (19), we expected to see more changes in relationships among patients with PPDB compared to patients without. No such effect was observed.

#### Alcohol Problems Before Surgery

Although the majority of patients reporting some sort of substance use disorder after RYGB has not had such problems before (20), patients with a history of alcohol problems may carry a vulnerability which makes them prone to alcohol problems after the operation. In our data we observed for women a clear association between having had alcohol problems prior to the surgery and PPDB. No such association was seen for men. Considering the long period since the operation, some patients would likely develop alcohol problems independently of whether RYGB increases such predisposition.

#### Memory

Obesity and cognitive function is still insufficiently understood, but there seems to be impairments across several cognitive domains (21). For chronic heavy intake of alcohol, however, there is a well-established understanding of its negative association to cognition (22). Our study included a question whether the patients had experienced problems with their memory after the operation. There was no such association for men, while data indicates a possible association for women. Asking about memory, however, is problematic in several ways. Firstly, because in a scientific perspective memory conceptually consists of several domains while most people may associate the word memory with cognitive functioning in general (23). Secondly, a person’s own experience of cognitive functioning depends on his or her context. Unemployed patients may face fewer situations requiring good cognitive functioning. Hence, a segment of the patients may not have reflected on this matter which may have introduced a systematic bias in the material.

#### Falling and Bone Fractures

Studies suggest that alcohol consumption impacts bone mass turnover negatively (24). Further, patients experience increased fracture risk after RYGB, possibly due to nutrition-related deterioration in bone microarchitecture and strength (25). Combined with the increased tendency of serious fall-related injuries after RYGB (26), we tested for associations both to self-reported falls last 12 months and bone-fractures incurring after the operation. For men, there was an association between PPDB and the tendency of falling, but this was not seen in women. Concerning bone fractures, PPDB was not associated to more bone fractures.

#### Working Out Regularly

Working out on a regular basis may be an indicator of self-care with potential value for sustaining weight loss after bariatric surgery. The BAROBS study mapped the patients’ exercise habits and we wanted to see if there were any association between PPDB and reporting exercise on a weekly level. No such associations were observed for neither men nor women.

#### Had Professional Help for Mental Problems Last 12 Months

As alcohol use disorder often is comorbid with mental disorders (27), we also expected to find an association between PPDB and utilization of mental health services. We did not see any such association for men, but the data indicated that an association might exist for women. Traditional masculine norms may contribute to lower help-seeking behavior in men compared to women (28), but there is too much uncertainty whether this association exists in women in the first place.

To sum up, among the 12 variables we considered as likely covariates to PPDB, we could only demonstrate clear associations for two of them, namely having alcohol problems prior to the operation (women) and having had a fall the last year (men). If allowing for a more explorative perspective for hypotheses, we saw indications of associations between PPDB and not receiving information about the operation’s effect on alcohol (men), reporting problems with memory (women), and, having utilized mental health services (women). The latter associations did not reach the necessary statistical significance, but give directions for hypotheses for future research.

Basis for our analyses was the operationalization of PPDB as two or more variables that could indicate an alcohol problem for a patient after RYGB. A legitimate question is whether three or more indicator variables would have produced a more valid category of patients with PPDB. In numbers, this would have reduced the PPDB-category from 59 (10.8%) to 33 (6.0%) patients. Comparing to the 20.8% cumulative incidence of postsurgery onset alcohol use disorder symptoms 5 years after surgery reported earlier (2), our two-indicator threshold for categorizing patients with PPDB does not seem to overestimate the proportion of patients that may face alcohol problems 10-15 years after RYGB. Narrowing this further down by requiring three indicator variables would potentially also complicate the Chi^2^-analyses due to low expected cell numbers. Since this material entirely consisted of patients undergoing RYGB, another question is the relevance of these findings to sleeve gastrectomy. In an earlier study on registry data we did not find any difference in risks for postoperative diagnoses related to alcohol problems (29). This, together with the physiological similarities between RYGB and sleeve gastrectomy (30), suggests that our findings may have relevance also for patients undergoing sleeve gastrectomy.

## Conclusion

Screening for alcohol problems in the wake of bariatric surgery is advised, but existing screening tools such as the AUDIT does not take into consideration the altered pharmacokinetics of ethanol after RYGB. This explorative study points to different post-operative phenomena among bariatric surgery patients that may be related to problematic drinking behavior in this particular population, such as effective patient education, previous alcohol problems, mental problems, problems with memory, and falling. These phenomena may be considered when developing a screening tool for alcohol problems among bariatric surgery patients.

## Strengths and Limitations

Although problematic drinking behavior after bariatric surgery is a clinically relevant phenomenon, the concept lacks a proper definition. Both diagnostic interviews and in-depth qualitative interviews of the patients could have provided useful additional references to what would be the valid criteria of such behavior within this particular group of patients.

The explorative approach in this paper involved a high number of analyses. This raises the concern for alpha error inflation. The p-levels were adjusted to compensate for this.

On the other hand, the BAROBS represents a comprehensive set of long-term data after RYGB including data describing patients’ drinking behavior. However, a prospective study with data collected also before surgery would have made inferences about causal mechanisms possible.

## Data Availability Statement

The raw data supporting the conclusions of this article will be made available by the authors upon request, given the approval of the Data Protection Officer. Requests to access the datasets should be directed to jorunn.sandvik@stolav.no.

## Ethics Statement

The studies involving human participants were reviewed and approved by the Regional Committee for Research Ethics (REK 2017/1828 REK South-East B). The participants provided their written informed consent to participate in this study.

## Author Contributions

All authors contributed to conception and design of the study. CK performed the statistical analysis. MS wrote the first draft of the manuscript while CK and JS wrote sections of the manuscript. All authors contributed to the article and approved the submitted version.

## Funding

This work was supported by the Liaison Committee for Education, Research and Innovation in Central Norway and funding from all three local hospital trusts.

## Conflict of Interest

The authors declare that the research was conducted in the absence of any commercial or financial relationships that could be construed as a potential conflict of interest.
